# Cerebellar haemorrhage and atrophy in infants born extremely preterm with intraventricular haemorrhage

**DOI:** 10.1111/dmcn.16123

**Published:** 2024-10-20

**Authors:** Julia Buchmayer, Renate Fuiko, Patric Kienast, Sophie Stummer, Gregor Kasprian, Angelika Berger, Katharina Goeral

**Affiliations:** ^1^ Comprehensive Center for Pediatrics, Department of Pediatrics and Adolescent Medicine, Division of Neonatology, Pediatric Intensive Care and Neuropediatrics Medical University of Vienna Vienna Austria; ^2^ Department of Radiology, Division of Neuroradiology and Musculoskeletal Radiology Medical University of Vienna Vienna Austria

## Abstract

**Aim:**

To investigate the impact of cerebellar haemorrhage (CBH) and atrophy in infants born extremely preterm with intraventricular haemorrhage (IVH) on neurodevelopment at 2 years of age.

**Method:**

This retrospective case–control study included infants born at less than 28 weeks' gestation with IVH over a 10‐year period. CBH, along with the assessment of cerebellar size, using magnetic resonance imaging, were studied. The impact of injuries on neurodevelopmental outcome at 2 years' corrected age was conducted, using multivariable regression analysis for comprehensive evaluation.

**Results:**

In a cohort of 103 patients, 69 (67.0%) showed CBH with a median grade of 1 (interquartile range = 0–3). At the corrected age of 2 years, CBH was significantly associated with impaired cognitive and motor outcome. CBH emerged as an independent predictor of poor cognitive and motor development, as well as cerebral palsy. Cerebellar atrophy, affecting 30 (29.1%) infants, was linked to a significantly worse outcome across all domains. Conversely, an increase in cerebellar size was correlated with improved motor development.

**Interpretation:**

Infants born extremely preterm with IVH and concomitant CBH exhibited significant cognitive and motor impairment. The severity of developmental delay correlated with the grade of CBH. These findings hold potential to support the prediction of long‐term outcome and parental counselling.

## Graphical abstract

This study investigates the impact of cerebellar hemorrhage (CBH) and atrophy in extremely preterm infants with intraventricular hemorrhage (IVH) on neurodevelopment at two years of age. Findings reveal that CBH is a significant predictor of impaired cognitive and motor outcomes, with cerebellar atrophy further exacerbating developmental delays.

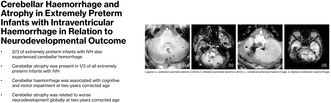

**This original article is commented by Cohen and Lerman‐Sagie on pages** 558–559 of this issue.

Plain language summary: https://onlinelibrary.wiley.com/doi/10.1111/dmcn.16167

**Abbreviations**
CBH: cerebellar haemorrhage
cMRI: cerebral magnetic resonance imaging
IVH: intraventricular haemorrhage
TCD: transverse cerebellar diameter
TEA: term‐equivalent age

**What this paper adds**

- Approximately two‐thirds of infants born extremely preterm with intraventricular haemorrhage also experienced cerebellar haemorrhage.
- Cerebellar atrophy was present in one‐third of all infants with intraventricular haemorrhage who were born extremely preterm.
- Cerebellar haemorrhage was associated with cognitive and motor impairment.
- Cerebellar atrophy was related to globally worse neurodevelopment.

Infants born extremely preterm, born at less than 28 weeks' gestational age, face a significant risk of experiencing cognitive, motor, learning, and behavioural disabilities,^1^, ^2^, ^3^, ^4^ despite increasing survival rates.^5^

Intraventricular haemorrhage (IVH), which has a multifactorial aetiology, is associated with high morbidity and mortality. It mostly originates from the germinal matrix in the lateral ventricles, which is particularly susceptible to fluctuating cerebral blood flow in high‐risk infants.^6^

Cerebellar haemorrhage (CBH) most commonly derives from the germinal matrix, which is additionally located in the fourth ventricle, the brainstem and the surrounding layer of the cerebellum, indicating potential shared risk factors with IVH.^7^, ^8^ Furthermore, large CBH can occur because of extension of intraventricular or subarachnoid blood into the cerebellum.^9^ Especially in the second half of pregnancy, the cerebellum undergoes rapid and substantial growth, making it particularly susceptible to growth restriction after injuries in high‐risk infants.^10^, ^11^, ^12^, ^13^

Bedside cerebral ultrasound is most commonly used for the identification and the grading of IVH in the neonatal intensive care unit.^14^ Nonetheless, brain injury, particularly haemorrhages, can be more sensitively described through cerebral magnetic resonance imaging (cMRI), which is commonly conducted around term‐equivalent age (TEA).^15^, ^16^

Abnormal cMRI findings at TEA have demonstrated a strong predictive association with adverse neurodevelopmental outcome,^17^, ^18^, ^19^ with Goeral et al.^20^ demonstrating this specifically in infants born preterm with IVH. In general, diagnostic sensitivity for the detection of CBH has been shown to be high using cMRI, ensuring reliable detection of haemorrhages located in the posterior fossa.^11^ As shown previously, cerebellar injuries are associated with impaired neurodevelopment in infants born preterm, particularly emphasizing compromised motor development.^21^, ^22^, ^23^

The aim of our present study was to evaluate the influence of CBH and atrophy in infants born preterm with IVH on neurodevelopmental outcome at 2 years' corrected age.

## METHOD

### Participants

This retrospective case–control study included infants born preterm admitted to a level IV neonatal intensive care unit at the Medical University Hospital in Vienna, Austria born at 22 + 6 to 27 + 6 weeks' gestational age with IVH grade I to IV from March 2011 to March 2021. IVH was graded according to the maximum extent of the haemorrhage on sequential cerebral ultrasound and routine cMRI. Neonates with central nervous system malformations, chromosomal anomalies, congenital malformations, metabolic disorders, and deceased patients were excluded.

The study population of infants born extremely preterm with IVH was divided into two groups: the case group, consisting of infants born extremely preterm who developed CBH; and the control group, where no CBH was visible on cMRI.

Perinatal and clinical variables were obtained, as described previously,^24^ including gestational age, mode of delivery, multiple birth, sex, birthweight, and respective centile, APGAR (Appearance, Pulse, Grimace, Activity, and Respiration), pH from both umbilical cord and first postnatal. Moreover, important neonatal diagnoses were collected: IVH, graded according to Papile et al.;^25^ white matter disease;^26^ CBH, graded according to Kidokoro et al.;^27^ blood culture‐proven sepsis; patent ductus arteriosus requiring both surgical or interventional closure; necrotizing enterocolitis above Bell's stage 2;^28^ severe retinopathy of prematurity defined as stage 3 and above according to the International Classification of Retinopathy of Prematurity,^29^ requiring intervention; and bronchopulmonary disease, defined as oxygen requirement for 36 weeks or longer,^30^ and analysed as potential risk factors.

### cMRI examinations

All cMRI scans were done as part of routine care at the local Department of Radiology, Division of Neuroradiology and Musculoskeletal Radiology, using a Philips Ingenia (Philips Healthcare, Best, the Netherlands) 1.5 T MR system. While in the past only patients with IVH grade II or higher were scanned, as of 2017, every infant born extremely preterm at less than 28 weeks' gestational age, regardless of any previously identified brain pathologies on serial cerebral ultrasound, was provided a cMRI scan at TEA. The large number of high‐grade IVH and impairment in our study population reflects this recent change in practice. All examinations were performed according to our local feed‐and‐wrap protocol using a vacuum immobilization mattress. Woodward et al.^19^ previously published a similar protocol.

### Cerebellar analyses

**FIGURE 1:**
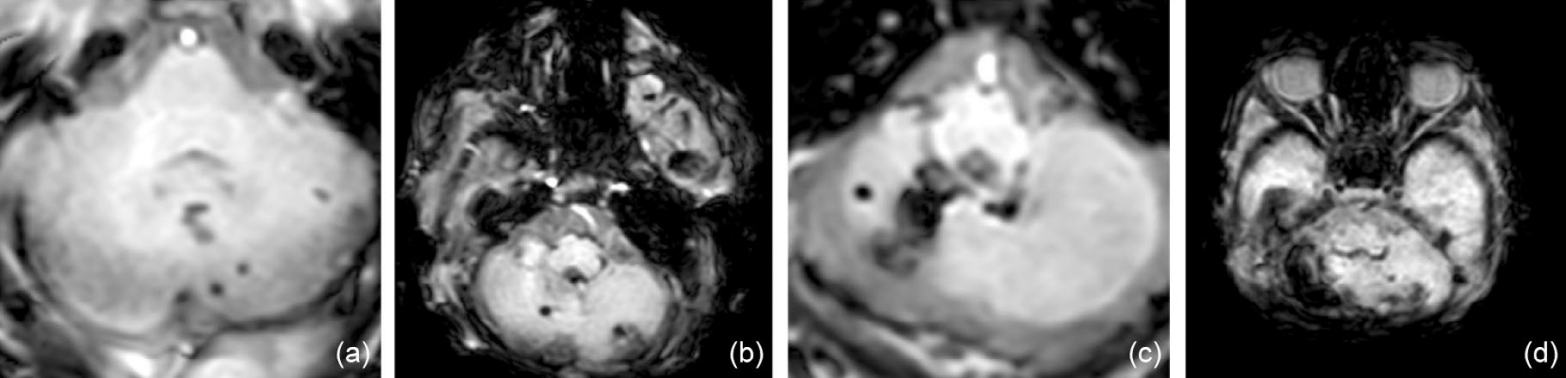
Classification of cerebellar haemorrhage. (a) Unilateral punctate lesions (≤ 3 mm). (b) Bilateral punctate lesions (≤ 3 mm). (c) Unilateral extensive haemorrhage (>3 mm). (d) Bilateral extensive haemorrhage (>3 mm).

Cerebellar analyses were conducted using the standard radiological DICOM program IMPAX EE (AGFA HealthCare, Mortsel, Belgium). CBH was graded according to Kidokoro et al.^31^ as shown in Figure 1, with grade I consisting of unilateral punctate lesions measuring less than 3 mm, grade II consisting of bilateral punctate lesions measuring less than 3 mm, and grade III consisting of a unilateral lesion measuring 3 mm or more; grade IV was diagnosed when extensive lesions were observed bilaterally. High‐grade injury was defined as CBH grade III or IV. Measurements of the extent of the haemorrhage were made on T2‐weighted sequences because the susceptibility‐weighted imaging sequence tends to overestimate the size of the bleeding. In addition, a category of ‘cerebellar atrophy’ was introduced, including atrophy of at least one hemisphere due to haemorrhage or intracranial pressure (e.g. trapped fourth ventricle). Cerebellar atrophy was defined as volume loss of at least one‐third of the hemisphere.^32^ Atrophy was graded using the definition by Bodensteiner et al.^33^ with four different patterns of injury as seen in Figure S1 (‘pancake’ cerebellum, damaged inferior cerebellum, diffuse cerebellar injury, and unilateral or focal injury). Additionally, cerebellar growth was categorized by measuring the transverse cerebellar diameter (TCD), which has been shown to correlate with cerebellar volume according to Nguyen The Tich et al.^34^

### Neurodevelopmental outcome

A neurodevelopmental follow‐up assessment was performed by trained clinical psychologists. Patients were tested using the Bayley Scales of Infant and Toddler Development, Third Edition^35^ at 2 years' corrected age using German norms; cognitive, language, and motor composite scores were obtained.

Cerebral palsy (CP) was classified using the Gross Motor Function Classification System (GMFCS) at 2 years' corrected age; a level was assigned. CP was grouped into ambulant CP (GMFCS levels I and II) and non‐ambulant CP (GMFCS levels III–V).

Motor function was assessed using the neurological examination from Amiel‐Tison et al.,^36^ which includes gross and fine motor skills, muscle tone, and reflexes.

### Statistical analysis

Analysis was performed using SPSS v23 for Mac (IBM Corp., Armonk, NY, USA). The distribution of data was assessed using the Kolmogorov–Smirnov test. Depending on the distribution, a Student's *t*‐test or analysis of variance, Mann–Whitney U or Kruskal–Wallis test, chi‐squared or Fisher's exact test, and odds ratios with 95% confidence intervals (CIs) were used for group comparisons. Univariate and multivariable linear regression analysis was used to examine the effect of CBH grade on the composite outcome scores. The multivariable model controlled for gestational age at birth and IVH grade to account for confounders. Our selection of control variables was guided by their association with CBH, their clinical relevance, and the need to avoid multicollinearity, ensuring robust and interpretable results. *p* < 0.05 was considered statistically significant.

Intraobserver and interobserver reliability were assessed for cMRI scoring on 2 subsequent days in 15% of patients; correlations coefficients were computed using a two‐way random model for absolute agreement and interpreted according to the strength of the agreement scale from Brennan and Silman.^37^

### Ethical approval

The study protocol received approval from the Institutional Review Board of the Medical University Vienna (EK 1677/2022). No written informed consent was obtained as cMRI is a standard routine examination at our neonatology department for all patients with IVH.

## RESULTS

### Study group

A total of 103 infants (male *n* = 67; 65%) were enrolled during the 10‐year study period. The median birthweight was 760 g (interquartile range [IQR] = 584–890); median gestational age at birth was 25 + 3 (IQR = 24 + 2–26 + 4) weeks. cMRI examinations were conducted at a median postmenstrual age of 38 + 1 weeks (IQR = 36 + 5–39 + 4). Comprehensive descriptive data are shown in Table 1.

**TABLE 1:** Neonatal and clinical characteristics.

Characteristic	cMRI (*n* = 103)	No CBH (*n* = 34)	CBH(*n* = 69)	*p*	OR (95% CI)
Gestational age at birth, weeks+days	25 + 3 (24 + 2–26 + 4)	25 + 3 (24 + 0–26 + 3)	25 + 3 (24 + 2–26 + 5)	0.577	–
22	1 (1.0)	0 (0)	1 (1.4)	0.471	–
23	15 (14.6)	7 (20.6)	8 (11.6)		–
24	24 (23.3)	7 (20.6)	17 (24.6)		–
25	25 (24.3)	9 (26.5)	16 (23.2)		–
26	17 (16.5)	5 (14.7)	12 (17.4)		–
27	21 (20.4)	6 (17.6)	15 (21.7)		–
Caesarean delivery	81 (78.6)	27 (79.4)	54 (78.3)	1.000	0.859 (0.315–2.339)
Multiple birth	33 (32.0)	14 (41.2)	19 (27.5)	0.427	1.842 (0.777–4.368)
Male	67 (65.0)	21 (61.8)	46 (66.7)	0.624	0.808 (0.344–1.897)
Birthweight, g	760 (584–890)	720 (602–875)	795 (582–900)	0.476	–
Birthweight centile	46 (26–62)	45 (27–58)	50 (26–65)	0.549	–
< 10th centile	6 (5.8)	2 (5.9)	4 (5.8)	1.000	0.985 (0.171–5.662)
APGAR score 1	7 (5–8)	7 (5–8)	7 (5–8)	0.422	–
APGAR score 5	8 (8–9)	9 (8–9)	8 (8–9)	0.356	–
APGAR score 10	9 (9–9)	9 (9–9)	9 (8–9)	0.082	–
pH_umb_	7.33 (7.24–7.36)	7.34 (7.31–7.36)	7.31 (7.19–7.36)	0.074	–
pH_neon_	7.18 (7.09–7.24)	7.21 (7.17–7.26)	7.14 (7.06–7.23)	0.025	–
Asphyxia	14 (13.6)	3 (8.8)	11 (15.9)	0.091	1.994 (0.517–7.688)
Blood culture—proven sepsis	38 (39.8)	14 (41.2)	24 (34.8)	0.416	0.701 (0.298–1.653)
IVH grade	3 (2–4)	2 (2)	3 (3–4)	<0.001	**–**
I	13 (12.6)	8 (23.5)	5 (7.2)	<0.001	–
II	26 (25.2)	18 (52.9)	8 (11.6)		–
III	34 (33.0)	2 (5.9)	32 (46.4)		–
IV (periventricular haemorrhagic infarction)	30 (29.1)	6 (17.6)	24 (34.8)		–
IVH bilateral	71 (68.9)	16 (47.1)	55 (79.7)	0.001	4.420 (1.809–10.796)
PHVD with neurosurgical intervention	35 (34.0)	3 (8.8)	32 (46.4)	<0.001	8.937 (2.495–32.016)
CBH	69 (67.0)	N/A	69 (100.0)	N/A	–
CBH grade	1 (0–3)		1 (0–3)		–
I	19 (18.4)		19 (27.5)		–
II	19 (18.4)		19 (27.5)		–
III	18 (17.5)		18 (26.1)		–
IV	13 (12.6)		13 (18.8)		–
Cerebellar atrophy	30 (29.1)		30 (43.5)		–
PDA with intervention	15 (14.6)	6 (17.6)	9 (13.0)	0.561	0.700 (0.227–2.159)
NEC with surgery	8 (7.8)	2 (5.9)	6 (8.7)	0.904	1.524 (0.291–7.981)
ROP with intervention	29 (28.2)	8 (23.5)	21 (30.4)	0.902	1.422 (0.553–3.654)
BPD (oxygen requirement ≥ 36 weeks)	43 (41.7)	13 (38.2)	30 (43.5)	0.612	1.243 (0.537–2.877)
Gestational age at cMRI, weeks+days	38 + 1 (36 + 5–39 + 4)	38 + 0 (37 + 0–39 + 0)	38 + 2 (36 + 5–40 + 0)	0.414	–

*Note*: Data are displayed as *n* (%) or median (IQR), as appropriate.

Abbreviations: APGAR, Appearance, Pulse, Grimace, Activity and Respiration; BPD, bronchopulmonary dysplasia; CBH, cerebellar haemorrhage; CI, confidence interval; cMRI, cerebral magnetic resonance imaging; IVH, intraventricular haemorrhage; NEC, necrotizing enterocolitis; OR, odds ratio; PDA, patent ductus arteriosus; pH_neon_, first neonatal pH after birth; pH_umb_, umbilical artery pH; PHVD, posthaemorrhagic ventricular dilatation; ROP, retinopathy of prematurity.

There were significant differences in neonatal and clinical characteristics between patients with and without CBH, for first neonatal pH as well as severity of intraventricular bleeding (IVH grade 3, IQR = 3–4 vs 2 [IQR = 2–2], *p* < 0.001; IVH bilateral 79.7% vs 49.1%, *p* = 0.001). Because of higher IVH severity in the group with CBH, a higher proportion of patients developed posthaemorrhagic ventricular dilatation requiring neurosurgical intervention (46.4% vs 8.8%, *p* < 0.001).

A total of 69 (67.0%) patients with IVH had CBH, with a median grade of 1 (IQR = 0–3). Atrophy of at least one hemisphere was found in 30 (29.1%) patients. Concerning different atrophy patterns, out of all infants with atrophy, three (10%) had a ‘pancake’ cerebellum, six (20%) had a damaged inferior cerebellum, eight (27%) had diffused cerebellar injury, and 13 (43%) had unilateral or focal atrophy.

Four of 34 infants without CBH exhibited small posterior fossa haemorrhage surrounding the cerebellum. Both intrarater and interrater reproducibility was classified as very good (≥0.81).^37^

### Neurodevelopmental outcome at 2 years' corrected age in children with cerebellar injury with the Bayley Scales of Infant and Toddler Development

Infants born extremely preterm with IVH and concomitant CBH had significantly lower cognitive scores (55 = IQR 55–80 vs 75 [IQR = 55–100], *p* = 0.005, *p*_adjusted_ = 0.059), similar language scores (60 [IQR = 45–75] vs 74 [IQR = 49–91], *p* = 0.087, *p*_adjusted_ = 0.091), and significantly lower motor scores (60 [IQR = 45–81] vs 84 [IQR = 67–92], *p* = 0.001, *p*_adjusted_ = 0.026), as shown in Table 2.

**TABLE 2:** Relationship between CBH and composite outcome scores, and cerebral palsy at 2 years' corrected age.

CBH	No CBH	CBH		*p*	*p*_adjusted_
Cognitive score	75 (55–100)	55 (55–80)		0.005	0.059
Language score	74 (49–91)	60 (45–75)		0.087	0.091
Motor score	84 (67–92)	60 (45–81)		0.001	0.026
Cerebral palsy	10 (29.4)	51 (73.9)		0.001	0.042
**CBH Kidokoro grade**	**No CBH**	**Low‐grade CBH**	**High‐grade CBH**	***p***	***p***_**adjusted**_
Cognitive score	75 (55–100)	65 (55–80)	55 (55–76)	0.009	0.102
Language score	74 (49–91)	66 (45–80)	59 (45–75)	0.191	0.208
Motor score	84 (67–92)	67 (48–82)	55 (45–73)	0.001	0.042
Cerebral palsy	10 (29.4)	25 (65.8)	26 (83.9)	<0.001	0.009
**Cerebellar atrophy**	**No atrophy**	**Atrophy**		***p***	***p***_**adjusted**_
Cognitive score	75 (55–90)	55 (55–56)		0.001	0.023
Language score	72 (48–86)	48 (45–70)		0.009	0.021
Motor score	79 (55–92)	45 (45–57)		<0.001	0.006
Cerebral palsy	36 (49.3)	25 (83.3)		<0.001	0.004

*Note*: Data are displayed as *n* (%) or median (interquartile range), as appropriate. *p*‐values were calculated using a Mann–Whitney *U* or Kruskal–Wallis test. *p*‐values were adjusted using univariate and multivariable regression analysis adjusted for independent variables (gestational age at birth, intraventricular haemorrhage grade). Abbreviation: CBH, cerebellar haemorrhage.

The extent of CBH was associated with the grade of developmental delay, mainly regarding motor development (cognitive: *p* = 0.009, *p*_adjusted_ = 0.102; motor: *p* = 0.001, *p*_adjusted_ = 0.042).

Cerebellar atrophy of at least one hemisphere was associated with significantly impaired composite outcome scores (cognitive: 55 [IQR = 55–56] vs 75 [IQR = 55–90], *p* = 0.001, *p*_adjusted_ = 0.023; language: 48 [IQR = 45–70] vs 72 [IQR = 48–86], *p* = 0.009, *p*_adjusted_ = 0.021; motor: 45 [IQR = 45–79] vs 79 [IQR = 55–92], *p* <0.001, *p*_adjusted_ = 0.006). There were no significant differences concerning the different shapes of cerebellar atrophy.

**FIGURE 2:**
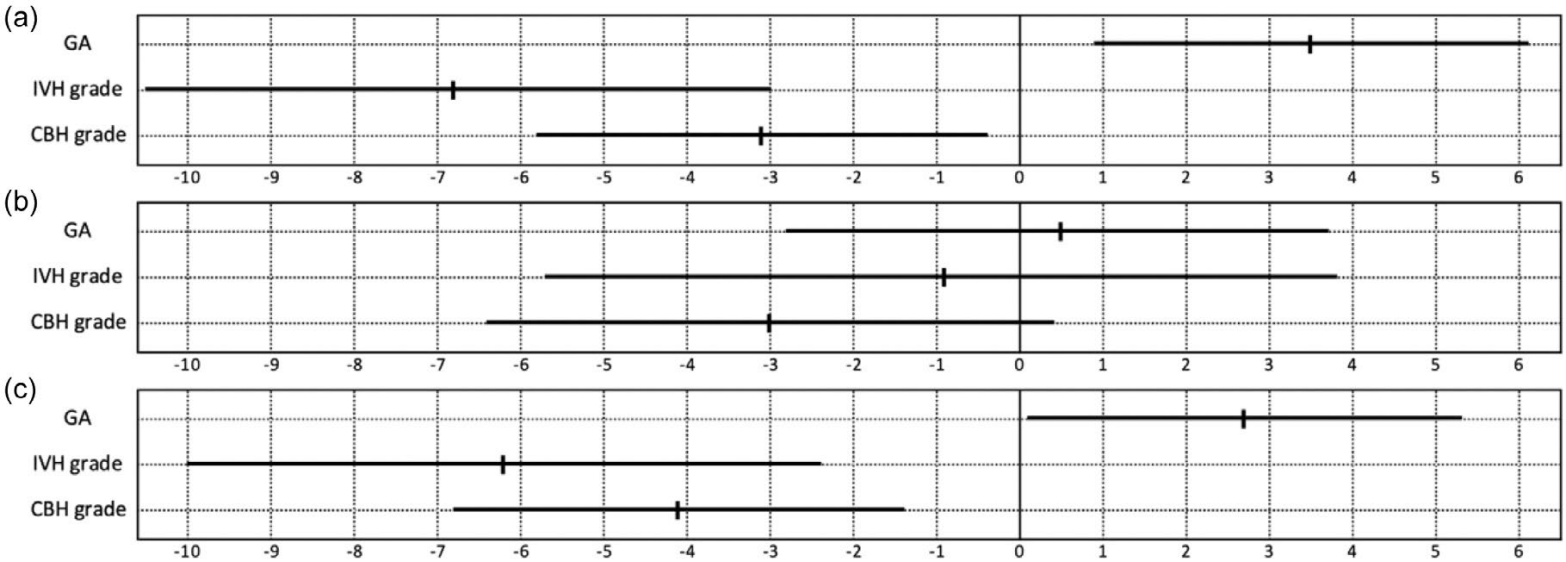
Forest plot representing unstandardized coefficients (*B*) along with their 95% confidence intervals of predictor variables on (a) cognitive, (b) language, and (c) motor outcomes calculated using multivariable regression analysis. *p*‐values were obtained using a multivariable regression analysis and included gestational age (GA) at birth, intraventricular haemorrhage (IVH), and cerebellar haemorrhage (CBH) grade: (a) gestational age at birth = 0.008, IVH = 0.001, CBH = 0.024; (b) gestational age at birth = 0.781, IVH = 0.696, CBH = 0.079; (c) gestational age at birth = 0.044, IVH = 0.002, CBH = 0.003.

Multivariable regression analysis confirmed an independent association of CBH with poor cognitive (*p* = 0.001, *p*_adjusted_ <0.001; language *p* = 0.042, *p*_adjusted_ = 0.119) and motor development (*p* < 0.001, *p*_adjusted_ <0.001). Figure 2 shows unstandardized coefficients (*B*) and their 95% CIs obtained using multivariable regression analysis, including the variables of gestational age at birth, IVH, and CBH grade. For cognitive outcome, standardized coefficients (Beta) revealed positive associations with gestational age at birth (+0.243, *p* = 0.008), and negative associations with IVH (−0.346, *p* = 0.001) and CBH grade (−0.221, *p* = 0.024). For motor outcome, coefficients were positive for gestational age at birth (+0.180, *p* = 0.044) and negative for IVH (−0.304, *p* = 0.002) and CBH grade (−0.284, *p* = 0.003).

Although gestational age at birth was similar between groups, we retained it in our visual representation (Figure 2) and analysis (Tables 2 and 3) as we previously demonstrated a notable impact of gestational age.^20^

**TABLE 3:** Univariate and multivariable regression results regarding outcome at 2 years' corrected age.

CBH Kidokoro grade	*B*	SEM	95% CI	Beta	*p*
Cognitive score	−4.638	1.363	−7.345 to −1.932	−0.331	0.001
Language score	−3.231	1.564	−6.337 to −0.125	−0.208	0.042
Motor score	−5.538	1.321	−8.159 to −2.917	−0.387	<0.001
**Adjusted model**	***B***_**adjusted**_	**SEM**_**adjusted**_	**95% CI**_**adjusted**_		***p***_**adjusted**_
Cognitive score	−6.364	2.660	−11.578 to −1.150		<0.001
Language score	−3.494	3.365	−10.090 to +3.102		0.229
Motor score	−7.541	2.693	−12.819 to −2.263		<0.001

*Note*: Models were adjusted for independent variables (gestational age at birth, intraventricular haemorrhage grade). Abbreviations: *B*, unstandardized regression coefficient calculated using a univariable model; *B*_adjusted_, unstandardized regression coefficient calculated using a multivariable model; Beta, standardized regression coefficient calculated using a univariable model; CBH, cerebellar haemorrhage; CI, confidence interval; SEM, standard error of the mean.

Infants without CBH but with small posterior fossa haemorrhage showed no significant differences in neurodevelopmental outcome (cognitive *p* = 0.980; motor *p* = 0.440; language *p* = 0.537).

### CP at 2 years' corrected age with cerebellar injury

Forty‐one neonates (39.8%) had an ambulant form of CP, while 20 neonates (19.4%) had a non‐ambulant form of CP. Infants born extremely preterm with IVH and concomitant CBH showed a significantly higher number of CP (51 [73.9%] vs. 10 [29.4%], *p* = 0.001, *p*_adjusted_ = 0.042). For infants with atrophy, there was a significant difference concerning CP (25 [83.3%] vs. 10 [29.4%], *p* < 0.001, *p*_adjusted_ = 0.002).

Furthermore, for different shapes of atrophy, we found significant differences regarding GMFCS level (*p* = 0.031). The most impaired outcome was seen for ‘pancake’ cerebellum (median GMFCS is IV [IQR = III–IV]), followed by diffuse cerebellar injury (GMFCS III [IQR = I–III]), damaged inferior cerebellum (GMFCS III [IQR = I–IV]), and unilateral or focal injury (GMFCS I [IQR = I–II]).

### Neurodevelopmental outcome with quantitative measurements

The median TCD was 45.4 mm (IQR = 41.8–48.3 mm), which was smaller compared to fetal MRI data from Garel^38^ at TEA with a TCD of 48.0 mm (IQR = 45.0–50.0 mm) and compared to our collective of infants born preterm at fewer than 28 weeks of gestation without morphological brain injury with a TCD of 48.1 mm (IQR = 45.9–49.9 mm). A larger TCD was significantly associated with better motor outcome scores (Pearson correlation coefficient [PCC] = 0.288, *p* = 0.004) but not better cognitive (PCC = 0.174, *p* = 0.091) or language outcome scores (PCC = 0.129, *p* = 0.214) at 2 years' corrected age.

## DISCUSSION

Our study evaluated the impact of CBH in infants born extremely preterm with IVH on standardized neurodevelopmental assessments at 2 years' corrected age. To our knowledge, this is the first study evaluating the influence of posterior fossa haemorrhage in infants born preterm with IVH, affecting 67.0% of the present cohort. Overall, there is a wide spectrum of lesions ranging from punctate or focal unilateral lesions to more extensive bi‐hemispheric haemorrhages or atrophy. Thus, this study provides further evidence about the importance of cerebellar injury in patients with IVH.

The responsibility of the cerebellum lies mainly in motor purposes, but the scientific literature increasingly highlights its significant role in affecting cognitive, learning, and behavioural functions in infants born very preterm. Limperopoulos et al.,^22^ Zayek et al.,^39^ Tam et al.,^40^ and O'Shea et al.^23^ found significantly impaired psychomotor and neurocognitive development in infants born preterm with cerebellar injuries. All these studies assessed the cerebellum using ultrasound. The sensitivity of cerebral ultrasound in detecting CBH was only one‐fifth (18%) compared to cMRI, which represents the criterion standard with a detection rate of 100%.^11^ In our study, a high detection rate was observed for high‐grade defects and cerebellar atrophy when using ultrasound. Those injuries were associated with a globally impaired outcome and CP in our cohort of infants born extremely preterm with IVH. Concerning the different shapes of cerebellar atrophy, an association with higher rates of CP for more severe defects was observed, which is consistent with scientific literature.^33^, ^41^ Supporting our findings, Calandrelli et al.^42^ showed that cMRI at TEA could predict long‐term motor outcome at 5 years of age in infants born extremely preterm.

Kidokoro et al.,^31^ who introduced the commonly used grading system for CBH, showed no significant differences in outcome between patients with or without cerebellar injuries. A notable strength of their study lies in its prospective nature, despite investigating a smaller number of patients (27 vs 69 patients with IVH), with most of their patients having low‐grade injury.

Chau et al.^43^ and Miller et al.^18^ reported similar findings, noting no association between CBH and neurodevelopment. The first study included only one infant with high‐grade IVH, while the second had only four patients with detectable CBH on cMRI at TEA. Both studies used no grading system for CBH. In our study, even low‐grade haemorrhages influenced the outcome, but as the size of the bleeding increased, scores got worse (Table 2).

In our study, focusing on the prevalence of CBH, 21% of all infants born extremely preterm born at fewer than 28 weeks of gestation, within a contemporary cohort following the implementation of routine cMRI over a 3‐year period, presented with CBH. Additionally, 69% of patients with IVH had concomitant CBH; this is consistent with the present analysis, which revealed a 67% prevalence of CBH.^24^ Compared to prospective cohorts examining more mature infants (≤ 32 weeks of gestation), Kidokoro et al.^31^ found a CBH prevalence of 23% in their St. Louis cohort; Steggerda et al.^44^ found a CBH prevalence of 19% and Garfinkle et al.^45^ found a CBH prevalence of 16%. The scientific literature indicates that infants born extremely preterm (< 28 weeks of gestation) exhibit higher rates of CBH compared to infants born between 28 weeks and fewer than 32 weeks of gestation.^46^

The existing scientific literature emphasizes the impact of supratentorial lesions, such as direct haemorrhage, or other concomitant factors, such as increased intracranial pressure, on cerebellar growth.^34^, ^47^ As two‐dimensional quantitative brain metrics represent cerebral volume,^38^ Tam et al.^40^ showed that cerebellar volume loss was associated with IVH, reflecting similar origins of damage. Overall, impaired cerebellar growth has been strongly linked to an adverse neurodevelopmental outcome.^34^, ^47^ This is consistent with our analysis, where a smaller TCD was associated with significantly lower motor outcome scores at 2 years' corrected age. When compared with fetal data, infants with morphological brain injury exhibited smaller cerebellar measurements.^38^ This is an important finding as it underscores the potential importance of neurodevelopmental outcome data for prenatal counselling of the parents of fetuses with cerebellar injuries.^48^

The presence of blood surrounding the cerebellum in absence of direct cerebellar parenchymal haemorrhage suggests a different pathogenesis from CBH but does not significantly alter the prognosis of infants born extremely preterm with IVH.

We did not find many neonatal or demographic risk factors for CBH in this high‐risk collective with IVH, except for the first neonatal pH and other neurological pathologies. Cerebellar damage has been associated with supratentorial injury, and the scientific literature supports that major early postnatal complications, such as altered haemodynamic factors, trauma, or coagulopathy are significantly associated with CBH.^24^, ^49^ Overall, there was a correlation between IVH and CBH grade, which supports the hypothesis of a similar pathophysiology.

### Strengths and limitations

The primary strength of our study lies in the extensive cohort of infants born extremely preterm with IVH, including both low‐grade and high‐grade cerebellar injuries. Their effect on the outcome could be evaluated, as availability of a neurodevelopmental outcome was a prerequisite for inclusion.

Concerning limitations, our collective only includes patients with IVH, but a contemporary matched control group is needed to further assess risk factors. The underclassification of neurodevelopmental delay with the Bayley Scales of Infant and Toddler Development, Third Edition is often discussed; therefore, we used population‐specific German norms, where this does not apply.^50^, ^51^ We used the well‐established CBH classification system developed by Kidokoro et al.,^27^ which primarily considers the size of the haemorrhage and the affected hemisphere, while not incorporating detailed anatomical localization, thus possibly overlooking the vermis or cerebellar lobules. Moreover, at our hospital, routine cMRI are conducted using a 1.5 T machine, whereas in some facilities a 3 T cMRI is considered the standard. Concerning the neurodevelopmental outcome, we did not explicitly assess cerebellar symptoms, such as ataxia or spasticity, as the cause of non‐ambulatory CP in our study. This major limitation is important to consider when interpreting our findings.

### Conclusion

Approximately two‐thirds of all infants born extremely preterm with IVH showed concomitant CBH. CBH was associated with significantly worse cognitive and motor outcome scores at 2 years' corrected age and was confirmed as an independent predictor using multivariable regression models. Cerebellar atrophy was linked to significantly impaired neurodevelopment across all domains, while increasing cerebellar size was associated with better motor outcome. Overall, this study has the potential to aid in the early identification of infants at significant risk for neurodevelopmental delay, may support prediction of long‐term outcome and parental counselling in patients with IVH, and facilitate timely therapeutic interventions.

## CONFLICT OF INTEREST STATEMENT

The data that support the findings are available on request from the corresponding author. We have no conflict of interest to declare.

## Supporting information


**Figure S1:** Different shapes of cerebellar atrophy on coronal cMRI.

## Data Availability

The data that support the findings of this study are available on request from the corresponding author.
