# Supplementary figures and images for: Cerebellar haemorrhage and atrophy in infants born extremely preterm with intraventricular haemorrhage
Source: Dev Med Child Neurol. 2024 Oct 20;67(5):609–17. doi: 10.1111/dmcn.16123 (PMC11965970; doi:10.1111/dmcn.16123)

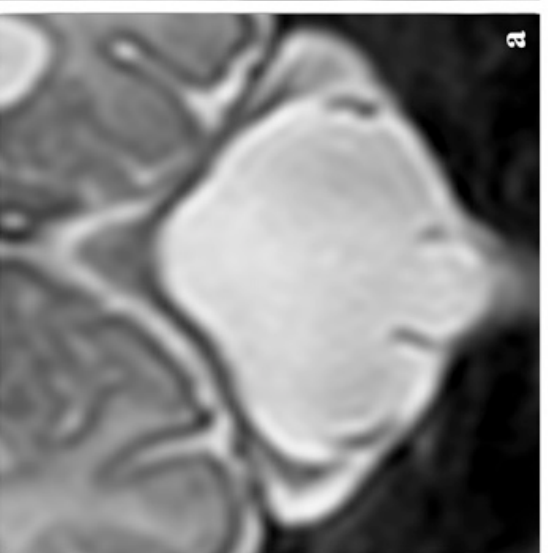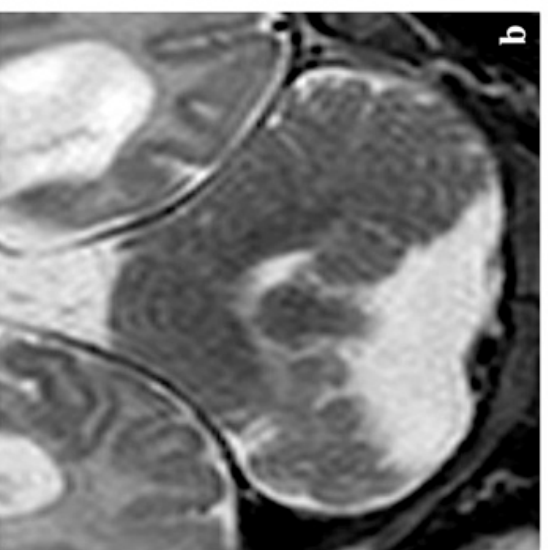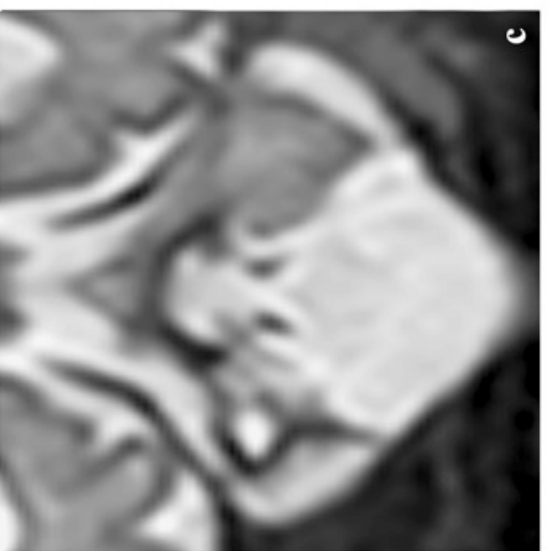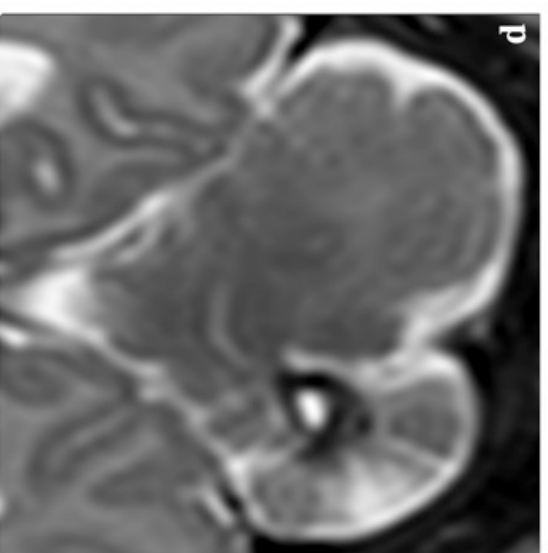

Supplement: Supplementary file 1 — Figure S1: Different shapes of cerebellar atrophy on coronal cMRI. [file DMCN-67-609-s001.pdf]
